# Comprehensive simulations of intracellular electric fields during exposure to tumor treating fields

**DOI:** 10.3389/fonc.2025.1520504

**Published:** 2025-05-28

**Authors:** Kaida Liu, Ping Dai, Zirong Liu, Haohan Fang, Xing Li, Wei Gao

**Affiliations:** ^1^ College of Automation Engineering, Nanjing University of Aeronautics and Astronautics, Nan Jing, Jiang Su, China; ^2^ Department of Radiotherapy, Shanghai Fourth People’s Hospital, School of Medicine, Tongji University, Shanghai, China

**Keywords:** tumor treating fields, intracellular electric fields, parameter effects, FEM, comprehensive simulations

## Abstract

Electric fields, as a unique physical form, have been widely used to manipulate and modulate biological processes. Tumor treating fields (TTFields) is one of the electro-therapy methods that deliveries intermediate frequency (100 kHz – 300 kHz), low intensity (1 V/cm – 3 V/cm) sinusoidal alternating current (AC) electric fields to inhibit tumor cell growth. When the tumor cells are exposed to TTFields, intracellular electric fields distribution will be a crucial clue for evaluating therapeutic effects and revealing mechanisms. This work systematically studied the TTFields distribution penetrating into the tumor cells by finite element method (FEM) simulations. We analyzed and compared the effects of various variables on the intracellular electric fields, including TTFields parameters, cellular geometry, and cellular electrical properties. We found that TTFields frequency, cell radius, cell membrane permittivity, cytoplasmic conductivity have significant impacts on the strength of intracellular electric fields. The results can be helpful for revealing TTFields mechanisms, explaining optimal parameter selection and better TTFields protocol design for different tumor types.

## 1 Introduction

Electrical activities play a crucial role in the biological system, facilitating essential processes such as substances transportation (1) and signal transmission (2). These functions rely on the intricate electrical structures within cells, their interactions, and intercellular electrochemical communication. Therefore, using external electric fields to modulate the biological activities is a natural insight. When the biological system is subjected to different external electric fields, different biophysical effects elicited. For instance, direct current (DC) electric fields typically induce electrolysis around electrodes inserted into tissue (3). External low-frequency electric fields (< 300 Hz) are commonly employed to stimulate nerve, muscle, heart, and other tissues (4), due to inherent bioelectrical processes typically operate at extremely low frequencies encompassing phenomena such as nerve and heart tissue action potentials. As electric fields transition to higher frequencies (> MHz), thermal effects become prominent due to electromagnetic loss, often utilized in techniques such as microwave ablation and radiofrequency ablation for cancerous tissue (5). Previously, intermediate frequencies (kHz to MHz) were considered to have negligible biophysical effects. However, in the early 2000s, an Israel research group made a groundbreaking discovery that intermediate frequency electric fields exhibit inhibitory effects on cancer cell proliferation. They termed these electric fields “tumor treating field (TTFields)” (6).

TTFields represent a type of intermediate frequency (100 kHz - 300 kHz) and low-intensity (1 V/cm - 3 V/cm, peak value) sinusoidal alternating electric fields. These fields have the ability penetrate cell membranes and reach the interior of cells, facilitated by the capacitive property of the cell membrane. In clinical application, TTFields are generated by a portable power supply and delivered to the tumor region through insulated electrode arrays attached on the skin (7). Due to its low electric field intensity and non-invasive hardware setup, this technique is considered safe with minimal side effects (7, 8). The promising performance of TTFields in clinical trials has led to widespread belief in their potential as the fourth modality in cancer treatment (7). This belief was substantiated by approvals from the Food and Drug Administration (FDA) for TTFields as a novel technique for treating glioblastoma multiforme (GBM) and malignant pleural mesothelioma (MPM) (9, 10), these approvals prolong the survival of patients compared conventional treatment techniques effectively. In clinical treatment, the primary limitation of TTFields is mild to moderate dermatitis resulting from prolonged electrode contact with the skin. This adverse effect can be mitigated by temporary discontinuation or topical medication.

Encouraged by its promising clinical outcomes, Tumor Treating Fields (TTFields) therapy has spurred further clinical trials to evaluate its potential efficacy in treating thoracic and abdominal tumors, including pancreatic adenocarcinoma (PAC) (11), platinum-resistant ovarian cancer (PROC) (12), hepatocellular carcinoma (HCC) (13), and non-small cell lung cancer (NSCLC) (14). Although complete and systematic trial data remain unpublished, preliminary results indicate therapeutic potential, with enhanced efficacy observed when TTFields are combined with conventional chemotherapy. TTFields have emerged as a promising non-invasive physical therapy modality in oncology, demonstrating significant potential as a complementary approach to conventional cancer treatments.

While TTFields therapy holds promise in cancer treatment, the precise mechanisms underlying how electric fields inhibit tumor cell proliferation remain elusive, particularly at the basic biophysical level. Although the prevailing theory suggests that TTFields exert force and torque to disrupt the cytoskeleton and inhibit mitotic processes, this notion has been challenged by theoretical computations indicating that the force and torque of TTFields may be too weak to cause significant mechanical effects on the cytoskeleton (15, 16). To reveal mechanisms of TTFields, firstly, it is essential to elucidate the distribution of electric field intensity within cells when exposed to TTFields. However, direct measurement of electric fields in single cells is exceedingly challenging due to their extremely small geometries. Consequently, silicon analysis has emerged as a crucial research method to simulate electric field distributions within cells with near-realistic accuracy. Many of the existing studies on TTFields simulation often simplifies single cells into spherical shapes in 3D or circular shapes in 2D, overlooking the influence of various cell structural geometries and electrical parameters on electric field distribution (17, 18). As a result, simulation results may deviate from actual scenarios. While achieving complete replication of the physical environment within cells in simulations may be impossible, conducting comprehensive simulations of TTFields distribution in cells is vital to systematically assess the impact of various parameters on intracellular electric fields. Such study can help effectively summarize how different factors influence the intracellular electric field and ultimately enhance the accuracy of simulations.

In this work, we developed single cell models and conducted comprehensive simulations to investigate the distribution of intracellular TTFields using finite element method (FEM). We focused on evaluating the influence of various parameters on the electric field distribution, including cell shapes, cell size, TTFields frequency, electrical properties of cell membrane and cytoplasm. Based on the simulation results, we revealed that TTFields frequency, cell radius, cell membrane dielectric constant, cytoplasmic conductivity have significant impacts on the strength of intracellular electric fields. These findings are crucial for improving the accuracy of simulations and facilitating more precise analyses of TTFields mechanisms.

## 2 Materials and methods

To investigate the influence of various parameters on TTFields distribution within cells, it is impractical to examine every possible scenario. Thus, we began by building typical single cell models and assigning commonly used parameters based on literature findings. This allowed us to compute intracellular electric field distributions and analyze how cell shape affects the results. Subsequently, for improving computational efficiency without sacrificing much accuracy, we selected a circular 2D cell model and varied geometrical and electric parameters to investigate their individual effects on electric field simulation outcomes.

### 2.1 Typical models and parameters

**Figure 1:**
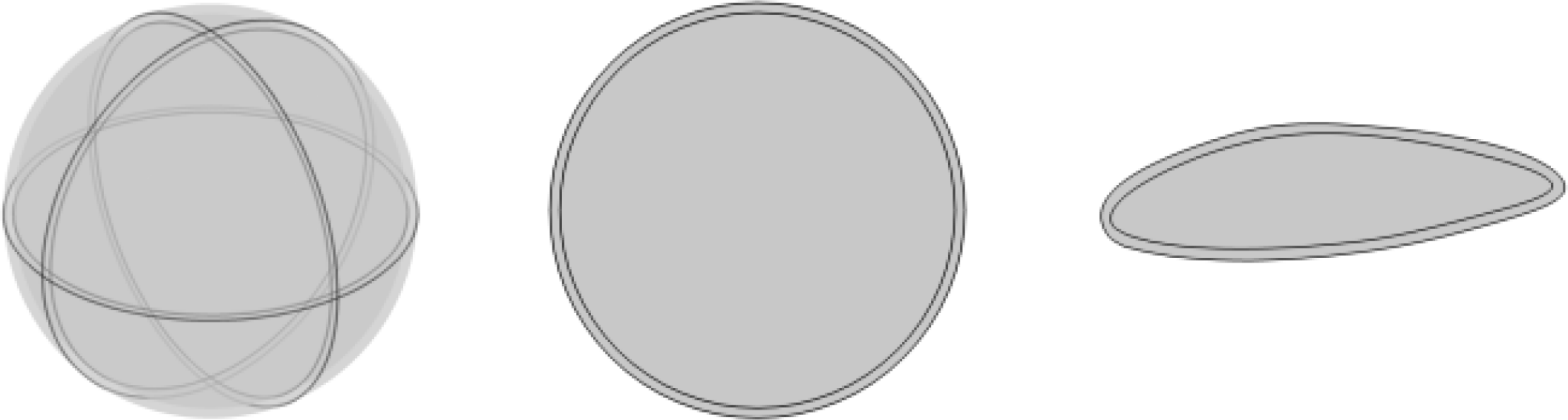
Typical simulation models of single cell.

We developed three typical single cell models: a 3D spherical cell model, a circular and an irregular spindle-shaped cell models in 2D, to investigate how shape and dimension influence TTFields distribution in the cell and to what extent. As depicted in **Figure 1**, the single cell represented with double-layered structures, consisting of the cell membrane and cytoplasm. Notably, we excluded the nucleus from the model as TTFields struggle to penetrate the nucleoplasm effectively. **Table 1** outlines the typical geometric and electrical parameters used in the models, all the parameters are selected based on previous studies which have been confirmed to be reasonable through experimental tests or clinical evaluation. It should be noted that in order to present the cell structure more clearly, we exaggerated the thickness of the cell membrane in the model figure, but in the actual simulation, the thickness of the membrane is consistent with the **Table 1**.

**Table 1:** Parameters of the models.

Parameters		models		
		A	B	C
Geometry (μm)	Radius or dimensions (19)	10	10	10×5
	Cell Membrane thickness (20)	5×10^‐3^	5×10^‐3^	5×10^‐3^
Conductivity (S/m)	Cell membrane (21)	10^-7^		
	Cytoplasm (22)	0.5		
	Extracellular medium (23)	0.6		
Relative Permittivity	Cell membrane (23)	8		
	Cytoplasm (23)	80		
	Extracellular medium (23)	80		
	Permittivity of the vacuum	ϵ_0_ = 8.854×10^‐12^ F/m		

### 2.2 Electric fields computation

TTFields are considered intermediate frequency electromagnetic fields, with a wavelength of approximately 1 km, significantly longer than the scale of biological systems. Consequently, the wave-like characteristics of TTFields are typically disregarded. When biological tissue is exposed to TTFields, the electric field penetrates the cell region in the form of electric current. Based on the Maxwell equation:


(1) $$\nabla \times \text{H}=\text{J}+\frac{\partial \text{D}}{\partial t}$$

The divergence of the above Equation 1 can be derived as Equation 2,


(2) $$\nabla \cdot (\text{J}+\frac{\partial \text{D}}{\partial t})=0$$

Where, ***H*** is the magnetic field intensity, ***J*** and ***D*** are the current density and electric displacement vector, respectively.

The relationships between current density ***J***, electric field intensity ***E***, and electric potential φ are given by


(3) $$\left\{ \begin{array}{l} \text{J}=\sigma \text{E}\\ \text{E}=-\nabla \varphi \end{array}\right.$$

The boundary conditions and interface connection conditions are


(4) $$\varphi_{\Gamma }=V$$

(5) $$\left\{ \begin{array}{l} \varphi_{1}=\varphi_{2}\\ \sigma_{1}\frac{\partial \varphi_{1}}{\partial \text{n}}=\sigma_{2}\frac{\partial \varphi_{2}}{\partial \text{n}} \end{array}\right.$$

Where, Γ and *V* are the boundary of computation region and TTFields electrode voltage on the boundary, respectively; ***n*** is the normal unit vector of the interface, subscript 1 and 2 representing two sides of the interface.

### 2.3 Finite element method (FEM)

The Finite Element Method (FEM) is a powerful numerical technique for approximating solutions to partial differential equation boundary value problems. The methodology firstly discretize or mesh the geometrical domain into finite small elements, and then formulating approximate solutions within each element using interpolation functions through variational principles. This systematic approach transforms complex continuous problems into solvable discrete systems, making FEM particularly effective for analyzing coupled physical fields including electromagnetic phenomena, thermal distributions, etc.

In this paper, we employed the finite element method (FEM) software COMSOL 5.3 to solve the aforementioned Equations 3-5, enabling us to obtain the electric field distribution. In the FEM simulations, the single-cell model is positioned within a computation domain three times larger than the single cell itself, this extended domain is not depicted in **Figure 1**.

## 3 Results

In this section, we initially examined the effects of cell geometry and TTFields frequencies on the electric field distribution within the cytoplasm. Subsequently, we delved into the impacts of other cellular electrical parameters.

### 3.1 Effect of ell shape

**Figure 2:**
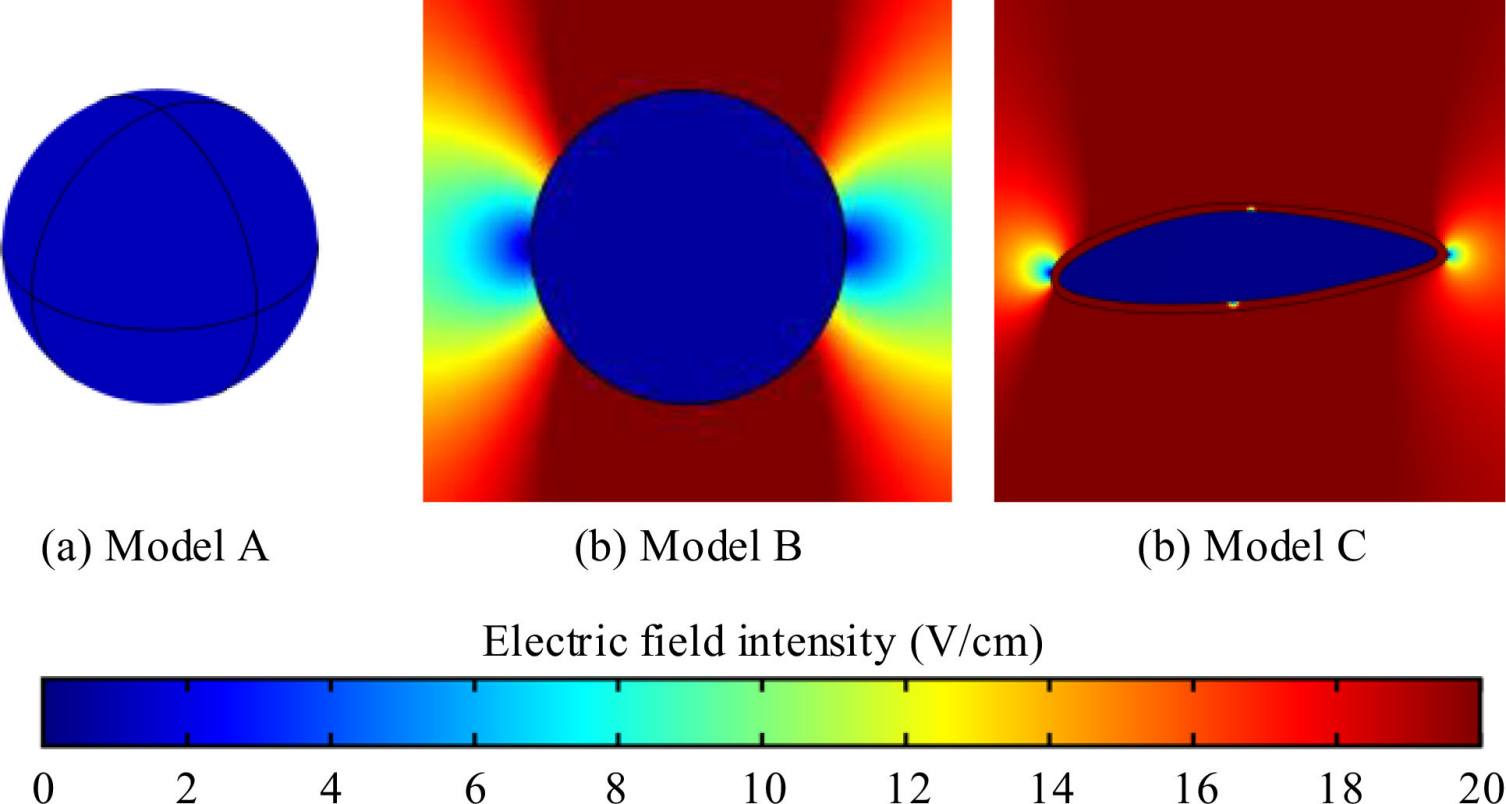
Electric field intensity distribution in different single cell models. **(a)** 3D spherical cell model, **(b)** 2D circular cell model **(c)** 2D irregular spindle-shaped cell model.

**Figure 3:**
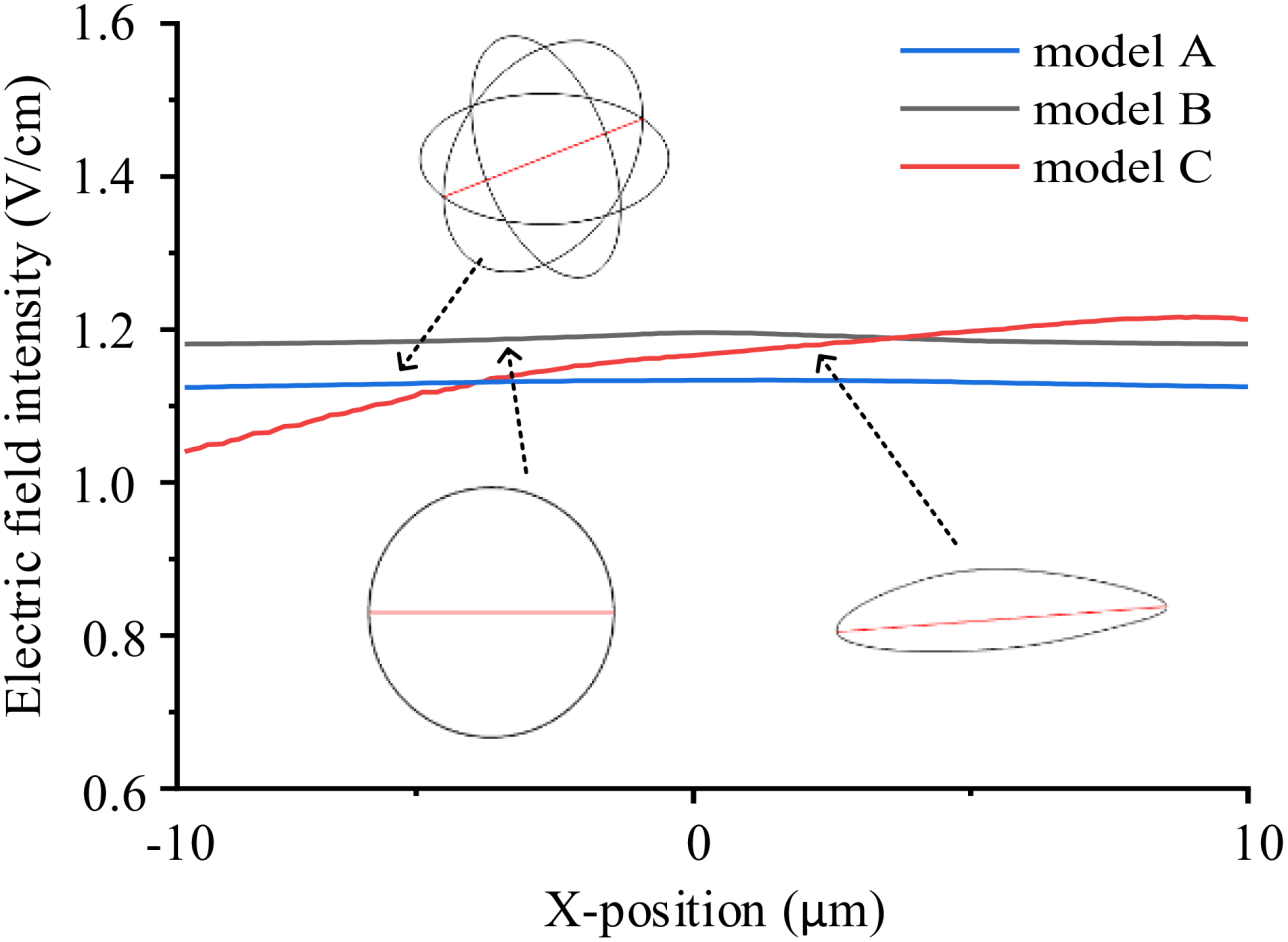
Electric field intensity distribution on the major axes (red lines in the model sketches) in different single cell models.

Cells exhibit diverse shapes depending on tissue types, including spherical, columnar, and spindle-shaped morphologies. In our study, we selected commonly encountered spherical and spindle-shaped single cells as representative examples to investigate the extent of influence that different shapes exert on electric field distribution. The single cell models and parameters are presented in **Figure 1** and **Table 1**. In the simulations exploring the impact of cell shapes, TTFields was set to a typical value of 2 V/cm (peak value) and a frequency of 200 kHz. The electric field distribution results are presented in **Figure 2**. To further compare the uniformity of the electric field in different cell models, we extracted the electric field values on the major axis in the cytoplasm and shown in **Figure 3**.

From the simulation results, **Figure 2** illustrates that the electric field intensity is stronger outside the cell than within the cytoplasm. This is due to the capacitance effect of the cell membrane, which has a strong shielding effect on medium and low-frequency electric fields. Across the three different cell models, the electric field intensity within the cytoplasm remains almost identical, at approximately 1.1 V/cm. Furthermore, as shown in **Figure 3**, TTFields are relatively evenly distributed inside the cell. These results indicate that variations in cell shape can indeed cause local uniformity in the internal electric field strength, but the impact on the overall electric field intensity is minimal. Consequently, we can infer that using a simplified 2D model will not significantly affect theoretical study results derived from the TTFields strength. Therefore, in the following sections, to improve computation efficiency, we will utilize model B to examine the effects of other parameters on the TTFields strength in the cytoplasm.

### 3.2 Effect of cell size

**Figure 4:**
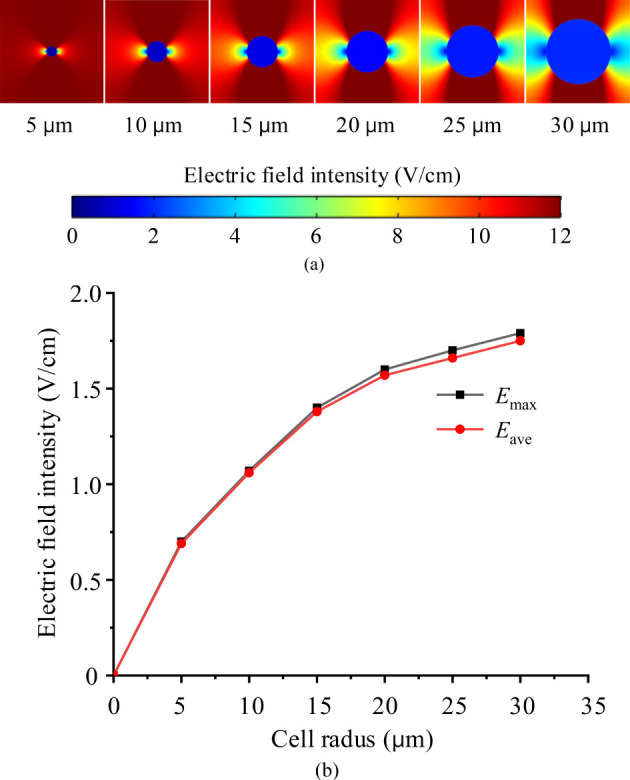
TTFields intensity in cytoplasm of various cell size, **(a)** electric field distribution maps, **(b)** maximum and average electric filed values.

Given that cell size varies across different tissues, the same external electric field may result in different electric field intensities within the cells. To evaluate whether cell size significantly affects TTFields distribution in the cytoplasm, we applied identical TTFields (2 V/cm and 200 kHz) across different simulation cases, varying only the cell radii, while keeping other parameters consistent as presented in **Table 1**. The simulation results, showing the electric field intensity distribution and the maximum values *E*_max_ and average values *E*_ave_ in the cytoplasm, are displayed in **Figure 4**.

Form the **Figure 4**, under identical external TTFields, a much stronger electric field is observed in the larger cell. This occurs because larger cells have a greater cell membrane surface area and, consequently, larger membrane capacitance and smaller impedance. As a result, a larger current passes through the cytoplasm when subjected to the same voltage. According to ***J*** =*σ* ***E*** (***J*** is the current density, *σ* is the conductivity, ***E*** is the electric field intensity), the electric field within the cytoplasm becomes stronger.

### 3.3 Effect of TTFields frequency

Subsequently, in the circular 2D single cell model, the external TTFields intensity setting remains unchanged as 2 V/cm, and the frequency range was adjusted from 50 kHz to 500 kHz, with increments of 50 kHz, to investigate the effects of electric field frequency.

**Figure 5:**
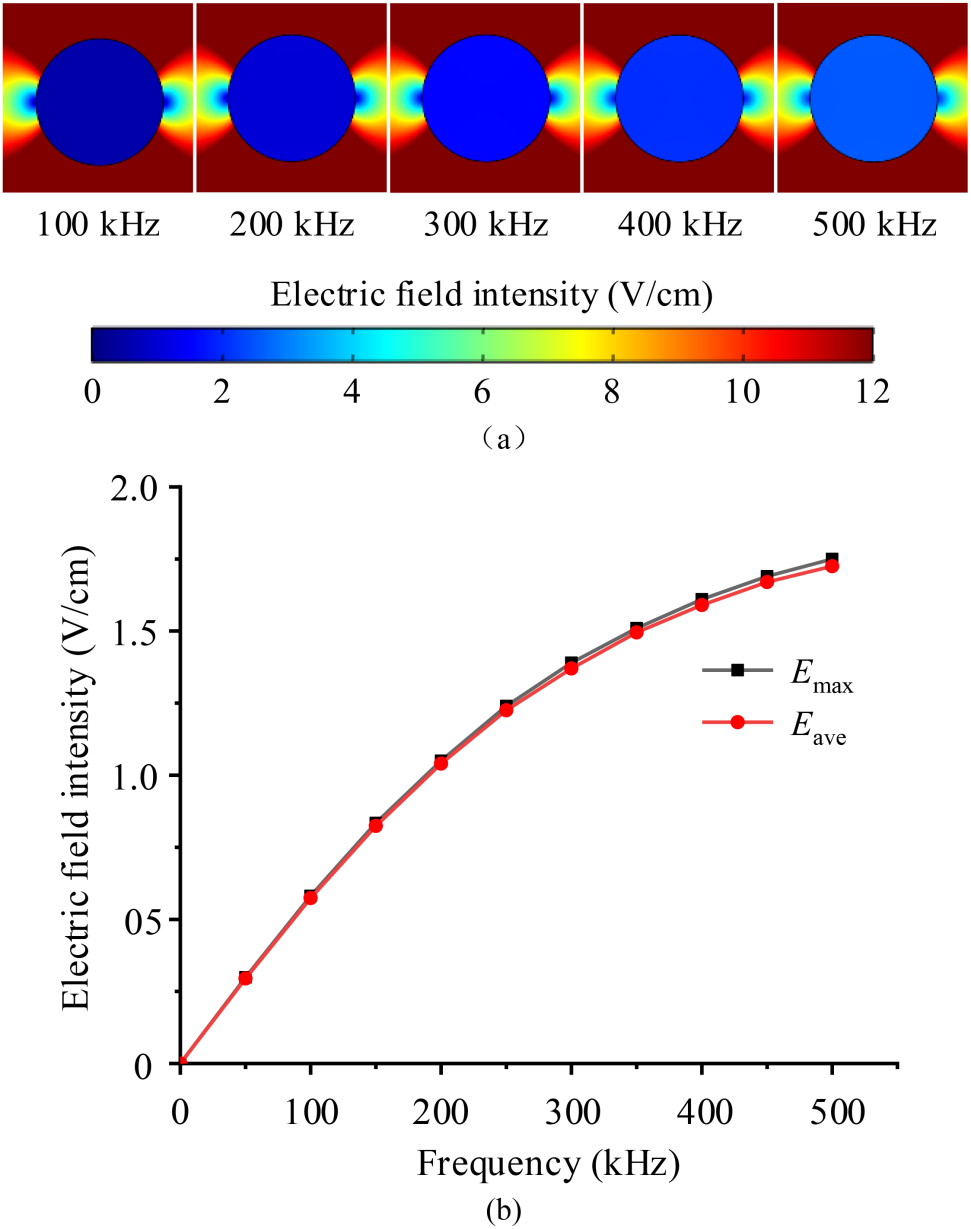
Frequency characteristics of TTFields intensity distribution in the cytoplasm, **(a)** electric field distribution maps, **(b)** maximum and average electric filed values.

**Figure 5** indicates that TTFields intensity in the cytoplasm increases with rising frequency, but the rate of this increase diminishes. This phenomenon occurs because, as the frequency increases, the capacitive impedance of the cell membrane decreases, allowing the current to penetrate the cytoplasm more easily and thereby increasing the electric field strength.

### 3.4 Effect of cell membrane conductivity and permittivity

**Figure 6:**
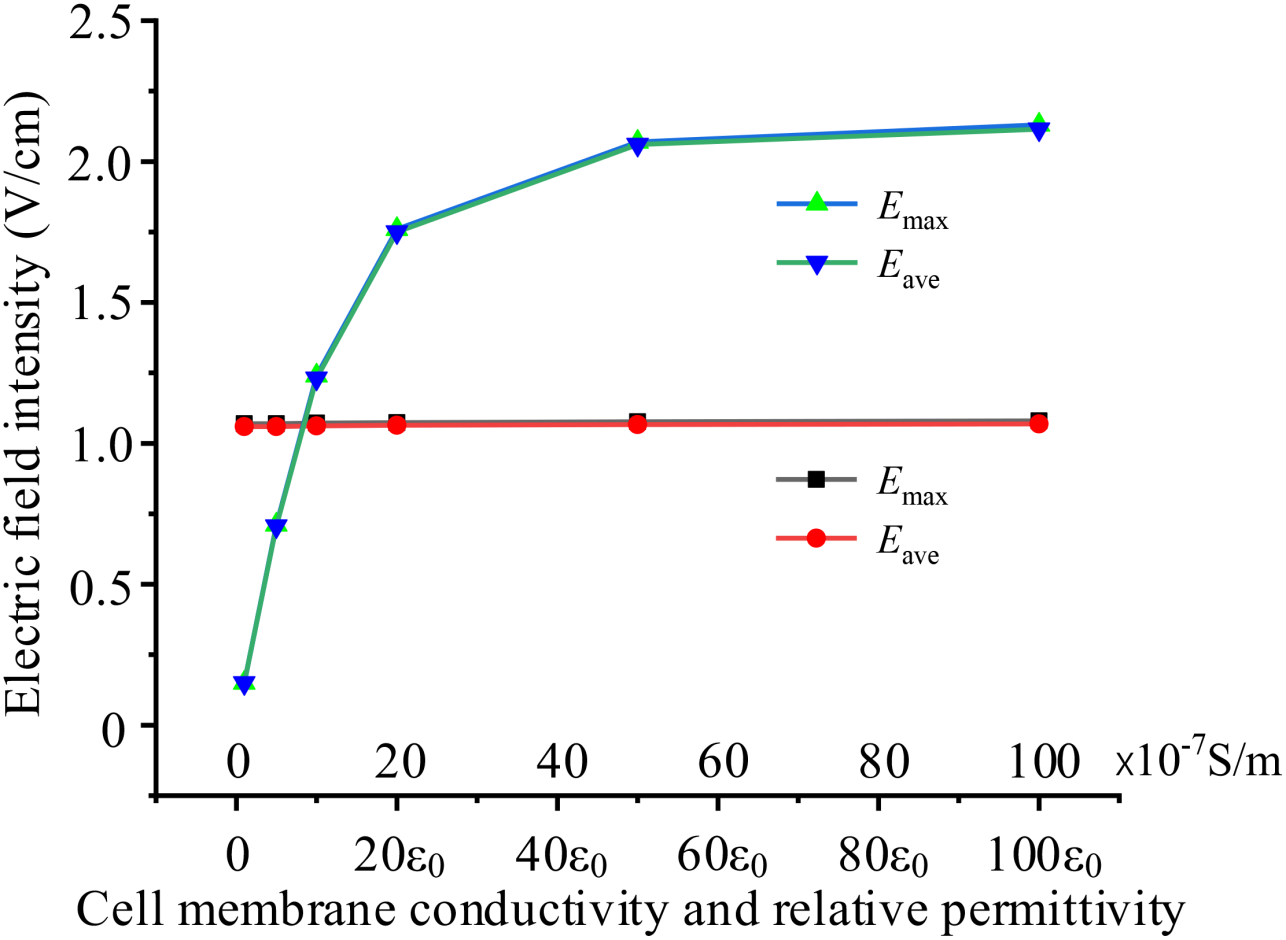
Effects of different cell membrane electrical parameters on intracellular electric field, black and red curves present the effect of conductivity, blue and green curves present the effect of permittivity.

When cells are exposed to external electric fields, the permeability of cell membranes may change (24), exhibiting differences in electrical parameters. Electric properties of cell membrane and cytoplasm affected by its permeability, could be dominate parameters to determine the strength of TTFields in the cytoplasm. In this subsection, based on the basic circular 2D single cell model, we examine the impacts by setting various membrane conductivity and permittivity. In all simulation cases, the external TTFields is 2 V/cm, 200 kHz. Additionally, we only change one variable (conductivity or permittivity) and the other parameters are shown in **Table 1**. For example, when investigate the effect of cell membrane conductivity, we set the conductivity range from 1×10^-7^ ~100×10^-7^ S/m, and keep the permittivity 8ϵ_0_ unchanged. Simulation results are presented in **Figure 6**.

**Figure 6** demonstrates that cell membrane permittivity significantly affects the electric field intensity in the cytoplasm, whereas cell membrane conductivity has almost no impact on the results. As cell membrane permittivity increases, the membrane capacitance also increases, leading to lower impedance and a larger current flow through the cell cytoplasm. Consequently, this results in a much stronger electric field within the cell.

### 3.5 Effect of cell cytoplasm conductivity and permittivity

**Figure 7:**
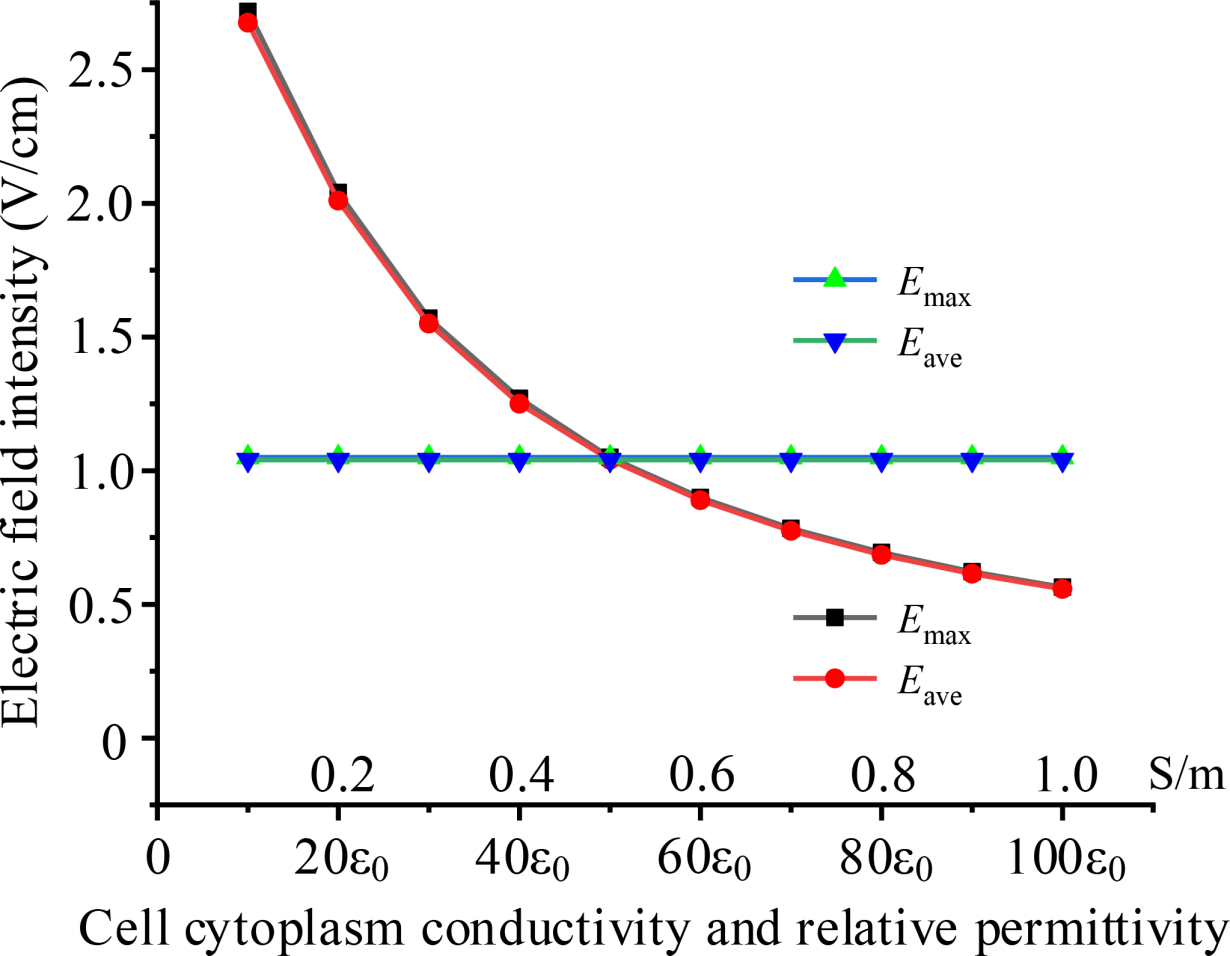
Effects of different cell cytoplasm electrical parameters on intracellular electric field, black and red curves present the effect of conductivity, blue and green curves present the effect of permittivity.

Different cells exhibit varying cytoplasmic electrical properties, which can lead to differences in electric field intensities within the cytoplasm when subjected to the same TTFields. Using the same model as in subsection D, the TTFields is 2 V/cm, 200 kHz, we simulated the electric field intensity in the cytoplasm under various conditions of cytoplasmic conductivity and permittivity. The simulation results are presented in **Figure 7**.

The results indicate that cytoplasmic conductivity significantly impacts intracellular electric field strength, with the electric field weakening as conductivity increases. In contrast, cytoplasmic permittivity has almost no effect on the results. This can be explained by the fact that, in the intermediate frequency range, the conductive properties of the cytoplasm are more dominant than its capacitive properties. Therefore, changes in conductivity have a greater influence on the electric field. Furthermore, when the cytoplasmic conductivity increasing, current is going up and a greater voltage drops on the cell membrane, resulting in lower electric field intensity in the cytoplasm.

## 4 Discussions

**Figure 8:**
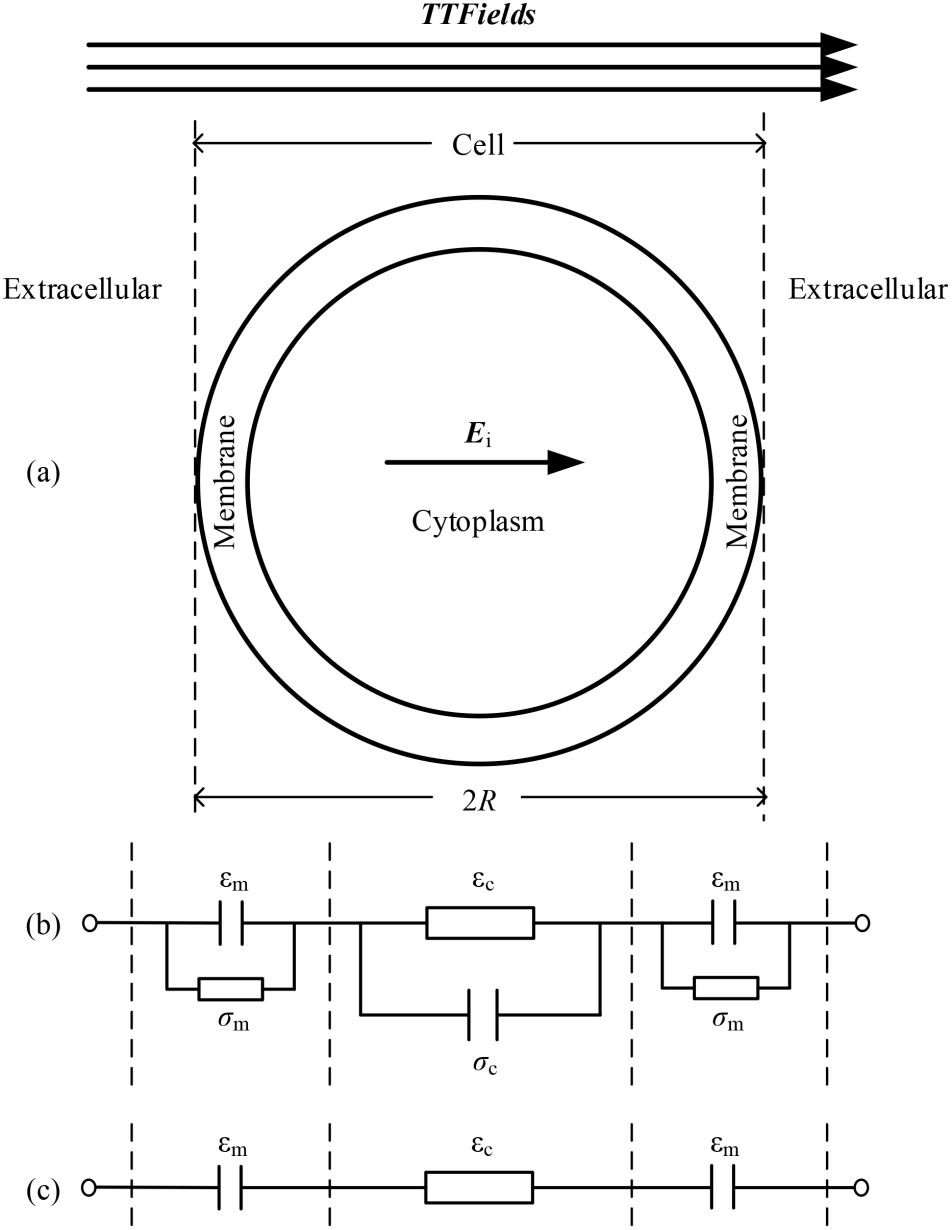
Single cell and its equivalent circuit model, **(a)** single cell model, **(b)** equivalent circuit model, **(c)** simplified equivalent circuit model under TTFields.

TTFields inhibits cell mitosis by applying voltage to generate an electric field inside the cell, and the strength of the inhibitory effect is related to the strength of the electric field in the cytoplasm. Due to the varying geometric and electrical parameters of cells, this article comprehensively simulated the influence of different parameters on the internal electric field of cells under the action of TTFields. The essence of TTFields is the current field effect, according to Ohm’s low ***J*** =*σ* ***E*** (***J*** is the current density, *σ* is the conductivity, ***E*** is the electric field intensity), the magnitude of the intracellular electric field is dependent on the current flowing through the cell. To analyze and verify the simulation results, we built the equivalent circuit diagram of a single cell illustrated in **Figure 8**. In the circuit model depicted in **Figure 8b**, the cell membrane and cytoplasm are modeled as a parallel connection of capacitance and conductivity.

When the cell is exposed to TTFields with a frequency around hundreds of kHz, the capacitive properties of the membrane and the conductive properties of the cytoplasm become dominant to determine the overall impedance. Consequently, the equivalent model can be simplified as shown in **Figure 8c**. Based on this circuit model, it is evident that parameters affecting the capacitance of the cell membrane and the conductivity of the cytoplasm significantly influence the electric field strength within the cell. Specifically, a larger cell size, higher frequency, greater cell membrane permittivity, and lower cytoplasm conductivity will enhance the electric field in the cytoplasm. These findings provide dual benefits: a) they advance theoretical understanding of TTFields’ biophysical mechanisms, and b) enable protocol optimization tailored to tumor-specific characteristics. By correlating field parameters with cellular geometry and electrical properties, treatment efficacy can be enhanced - for instance, applying intensified fields to smaller tumor cells may simultaneously inhibit proliferation and potentiate immune responses. This approach demonstrates how physical properties can guide precision TTFields therapy.

## 5 Conclusions

This study conducted comprehensive simulations to investigate the effects of various parameters on the intracellular electric field intensity when cells are subjected to TTFields. According to our simulation results, we found that in different models with varying cell shapes, the electric field intensities in the cytoplasm are nearly identical, with the exception of some local unevenness. Therefore, using a simplified 2D circular model in theoretical studies will not result in significant deviations, this model is reasonable used in the literatures or similar future theoretical research.

Furthermore, when the cell is exposed to TTFields, we observed that the intracellular electric field is highly sensitive to TTFields frequency, cell size, membrane permittivity, and cytoplasm conductivity. This conclusion may elucidate why TTFields inhibit tumor cells more effectively while having less impact on healthy cells. Our findings could be significant in helping to analyze the theoretical mechanisms of TTFields and in developing optimal TTFields parameters to inhibit different types of tumors.

## Data Availability

The original contributions presented in the study are included in the article/supplementary material. Further inquiries can be directed to the corresponding author.
